# Integration of ultra-high field MRI and histology for connectome based research of brain disorders

**DOI:** 10.3389/fnana.2013.00031

**Published:** 2013-09-27

**Authors:** Shan Yang, Zhengyi Yang, Karin Fischer, Kai Zhong, Jörg Stadler, Frank Godenschweger, Johann Steiner, Hans-Jochen Heinze, Hans-Gert Bernstein, Bernhard Bogerts, Christian Mawrin, David C. Reutens, Oliver Speck, Martin Walter

**Affiliations:** ^1^Department of Biomedical Magnetic Resonance, Otto-von-Guericke UniversityMagdeburg, Germany; ^2^Leibniz Institute for NeurobiologyMagdeburg, Germany; ^3^School of Information Technology and Electrical Engineering, University of Queensland, BrisbaneQLD, Australia; ^4^Institute of Anatomy, Otto-von-Guericke UniversityMagdeburg, Germany; ^5^High Magnetic Field Laboratory, Chinese Academy of SciencesHefei, China; ^6^Department of Psychiatry, Otto-von-Guericke UniversityMagdeburg, Germany; ^7^Center for Behavioral Brain Sciences (CBBS)Magdeburg, Germany; ^8^Department of Neurology, Otto-von-Guericke UniversityMagdeburg, Germany; ^9^German Center for Neurodegenerative Diseases (DZNE)Magdeburg, Germany; ^10^Institute of Neuropathology, Otto-von-Guericke UniversityMagdeburg, Germany; ^11^Centre for Advanced Imaging, University of Queensland, BrisbaneQLD, Australia

**Keywords:** 7 tesla, post mortem, biological psychiatry, high resolution imaging, translational neuroscience, 3D reconstruction, brain data base, connectome

## Abstract

Ultra-high field magnetic resonance imaging (MRI) became increasingly relevant for *in vivo* neuroscientific research because of improved spatial resolutions. However, this is still the unchallenged domain of histological studies, which long played an important role in the investigation of neuropsychiatric disorders. While the field of biological psychiatry strongly advanced on macroscopic levels, current developments are rediscovering the richness of immunohistological information when attempting a multi-level systematic approach to brain function and dysfunction. For most studies, histology sections lost information on three-dimensional reconstructions. Translating histological sections to 3D-volumes would thus not only allow for multi-stain and multi-subject alignment in post mortem data, but also provide a crucial step in big data initiatives involving the network analyses currently performed with *in vivo* MRI. We therefore investigated potential pitfalls during integration of MR and histological information where no additional blockface information is available. We demonstrated that strengths and requirements from both methods can be effectively combined at a spatial resolution of 200 μm. However, the success of this approach is heavily dependent on choices of hardware, sequence and reconstruction. We provide a fully automated pipeline that optimizes histological 3D reconstructions, providing a potentially powerful solution not only for primary human post mortem research institutions in neuropsychiatric research, but also to help alleviate the massive workloads in neuroanatomical atlas initiatives. We further demonstrate (for the first time) the feasibility and quality of ultra-high spatial resolution (150 μm isotopic) imaging of the entire human brain MRI at 7T, offering new opportunities for analyses on MR-derived information.

## Introduction

Rapid technical advances in magnetic resonance imaging (MRI) have strengthened the role of this important non-invasive method in neuropsychiatric research and significantly shaped neuroscientific questions in humans during the last 20 years. The most evident reasons for neuroimaging's rapid progression—a transition away from a field mainly characterized by histological observations in post mortem brain tissue, toward structural and functional characterizations *in vivo*—can be found in its capacity for experimental control of observations as well as its distinct advantages in allowing for longitudinal studies. In addition to applications for functional studies, these advantages also have the potential to produce fundamental insights regarding anatomical variations in the progression of brain disorders or consecutive clinical and psychological characterizations associated with aberrant brain anatomy.

Post mortem histology has seemingly lost its premier role in biological psychiatry, despite a number of important insights that remain inaccessible to investigations based on MRI. Most importantly, gross changes in volumetry have cytoarchitectonic aspects based on individual contributions of different cell populations, which are crucial for characterizing underlying pathology. The cellular origins of volume loss (e.g., glial vs. neuronal) are not visible by corresponding differences in contrast, and as such remain the domain of histological investigations, which have the advantage of numerous immunohistochemical approaches to stain cell types according to transmitter types, receptor subtypes, and expression of specific enzymes or immunological activation (Stockmeier et al., 2004). A second advantage is higher spatial resolution, which in addition to immunohistological differences, allows for investigations of substructures for which MRI has insufficient spatial resolution.

The increasing availability of higher field strengths (e.g., 7T magnets) challenges the historic primacy of histological studies by increasing spatial resolutions. Stronger fields allow for a higher signal to noise ratio (SNR) at more detailed spatial resolutions, thereby improving contrast mechanisms such as phase shift (Shmueli et al., 2009; Yao et al., 2009) or other tissue specifics in magnetic resonance (MR) signals (Mikawa et al., 2001; Rohrer et al., 2005).

MR microscopy recently reached 6.25 μm in-plane resolution for small sections of brain tissue (Flint et al., 2012). Ultra-high field MRI investigations in post mortem tissue thus reached the level of laminar specification of functional subunits, such as identifying granular and agranular layers in primary visual or motor cortices (Naito et al., 2000; Binkofski et al., 2002). Similarly, the functional segregation of previously indistinguishable subunits of subcortical regions (e.g., thalamic nuclei) became possible even *in vivo*, based on either specific MR signatures (Hoffmann et al., 2009; Kanowski et al., 2010) or high functional resolutions (Walter et al., 2008). However, direct confirmation of anatomical correspondences remains missing for most of the postulated identifications, primarily because several unmet technical needs remain in establishing correspondences between histoarchitectonic and MR-based information in the same brain. Firstly, and most importantly, a direct correspondence of brain tissue accessible by MRI and subsequently prepared histological observations is normally lost due to shrinkage during preparation of histological sections. Secondly, for the same brain, individual histological sections lose their anatomical correspondence to each other in three dimensions (two translational and one rotational), even if each section represents a consecutive follow up observation stacking on top of the previous section.

Reconstruction by simply stacking registered histological sections does not yield results of sufficient quality due to inter-section misalignment and intra-section distortions caused by histological processing (Yang et al., 2012b). The latter introduces non-linear tissue distortions, such as separation, missing parts, squashing, stretching, folding and tearing, as well as artifacts including air bubbles and dust (Ju et al., 2006). Intensity inhomogeneity within and across sections caused by inconsistent staining and uneven illumination are other common issues (Malandain and Bardinet, 2003; Malandain et al., 2004; Chakravarty et al., 2006). These problems not only limit histological verification of hypotheses of anatomical correspondence of MR-based parcellations, but also hamper the reconstruction of histological information into 3D space.

Reliable and valid volumetric reconstructions of histological sections are necessary for future investigations that concern immunohistological information unique for post mortem analyses. This is especially important if alignment to a standard space (e.g., MNI or Talairach spaces) is intended (Brett et al., 2004). Such normalization steps, which are common in most MR-based approaches, would strongly improve generalizability of histological findings on a group level. Further, for the first time, it would be possible to spatially integrate multimodal information derived from both post mortem and *in vivo* observations. As a consequence, large-scale investigations in 3D space—such as network analyses of histological entities like inhibitory interneurons—would be easily possible using the types of digital image processing algorithms that have been successfully utilized for MRI.

Methods of histological volume reconstruction reported in the literature can be broadly categorized into two groups: either without a spatial reference containing inter-section geometric information (Kim et al., 1997; Ourselin et al., 2001a,b; Chakravarty et al., 2006; Pitiot and Guimond, 2008), or including this information (Yushkevich et al., 2006; Yang et al., 2012b). An inherent drawback of the former category of methods is that good inter-section registration does not guarantee that overall brain shape will be accurately reconstructed. More specifically, error accumulation can result in the so-called “banana effect”: a linear structure being reconstructed as a spatial curve (Beare et al., 2008). Smoothness-driven registration methods were proposed for achieving visually plausible volume reconstructions with continuous structures (Wirtz et al., 2005; Badea et al., 2007; Cifor et al., 2009, 2011), but their ability to rectify the “banana effect” is unknown. In the latter category, fiducial markers (Bardinet et al., 2002; Yelnik et al., 2007), block-face imaging (Schormann and Zilles, 1998; Bürgel et al., 1999; Denk and Horstmann, 2004; Meyer et al., 2006; Dauguet et al., 2007; Schmitt et al., 2007; Chakravarty et al., 2008; Uberti et al., 2009), and MRI images (Malandain et al., 2004; Yushkevich et al., 2006; Yang et al., 2012b) were used as spatial references. Recently, Amunts' study demonstrated an ultra-high resolution 3D human brain model based on blockface imaging (Amunts et al., 2013). Blockface imaging guides the reconstruction process with a spatial reference based on a volume formed by stacking the digitized images of the surface of the remaining tissue block during histological sectioning (Schormann and Zilles, 1998; Dauguet et al., 2007; Uberti et al., 2009). Because the immobility of the block maintains the spatial relationship between adjacent sections, it serves as the “ground truth” for creating the histology volume. However, acquisition of block-face images could complicate histological processing because of the extra equipment and handling required. Non-linear registration is still needed to warp histological sections to MRI. In this article we describe the beneficial effects of introducing ultra-high resolution MRI in order to obtain increased degrees of freedom for non-linear transformations.

For the first time, ultra-high resolution imaging via high-field MRI scanners can match histological data at a sub-millimetre scale of 100–300 μm (Yang et al., 2011, 2012a). This resolution is currently achieved only in comparably long scans of fixated brains, which do not require histological preparation for the same isotropic resolution. However, while histological sections would in principle allow through-plane resolutions of up to 20 μm, the inclusion of multiple histochemical characterizations and limited availability of the specimens—especially if taken from patients—normally results in staining of every 10th–15th section, thus resulting in an effective section thickness similar to high resolution MRI slice thickness. Therefore, neuropsychiatric research enjoys a new possibility for merging these traditionally distinct research approaches. As a consequence, histological targeting of contrasts between gray and white matter (WM) would offer supreme advantages for estimations of reduced cortical thickness in predefined regions (even at the same spatial resolution), or on a whole brain level. Additionally, 3D approaches to such materials would further allow for inter-subject alignment based on gyrification. Furthermore, MR-based parcellation of whole brains before histological preparations would offer the potential for improved estimations of non-linear, tissue specific shrinkage during paraffin embedding, or for misalignment of split parts that frequently happen during the sectioning (Yang et al., 2012b).

We examined the potential of post mortem MRI in a multimodal framework that explicitly aims at combining information from MRI and histology on the same specimens. We explored a major advantage of this approach for MR guided volumetric reconstruction of histological sections to overcome current difficulties of traditional post mortem research.

**Figure 1:**
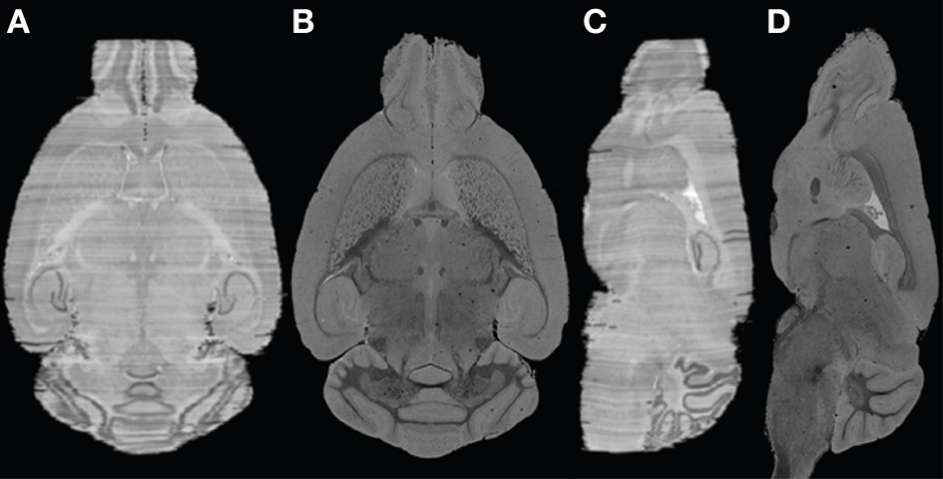
**Example of histological volume reconstructed for the mouse brain. (A)** and **(C)** axial and coronal views of the constructed histological volume; **(B)** and **(D)** the corresponding MR slices.

In a previous investigation, MR images and histological sections of a C57BL/6J mouse (12-week old male) brain were obtained as part of building an anatomical atlas (Yang et al., 2012b). 3D MR images were reconstructed at 30 μm in each dimension. Subsequently, more than 350 Nissl-stained sections of 40 μm section thickness and in-plane resolution 65 × 65 μm^2^ were acquired. Given the high quality of the MRI data, iterative reconstruction of post mortem MR and histology information of the mouse brain yielded a high quality overlay of both modalities, as shown in Figure 1. One major remaining problem was the variable intensities observed from section to section, visualized as stripes, which could affect the volumetric reconstruction. In this study, the efficacy of the histology reconstruction method was therefore validated on human post mortem brains, targeting both known and specific problems of MR-guided 3D reconstruction of human brain sections.

## Materials and methods

### Image acquisition

Two formalin-fixated human post mortem brains were scanned using a 7T whole body MRI scanner (Magnetom 7T Siemens, Germany, Erlangen). The first brain (76-year-old female), tagged as HB1, consisted of the middle 1/3 of the whole brain representing the histological middle block ranging from the anterior to posterior part of the corpus callosum and thus encompassing all subcortical structures. Anatomical MRI was acquired using Fast Low Angle Shot (FLASH) sequence with a 24 channel coil and the following parameters: *TR* = 28 ms, *TE* = 18 ms, flip angle = 11°, bandwidth = 80 Hz/pixel, resolution = 200 × 200 × 300 μm^3^, 10 averages and total acquisition time = 40.3 h. Subsequently, the brain underwent standard processing for histology leading to paraffin embedded blocks. After microtome preparation and deparaffination, every 10th section (20 μm) was stained for Nissl (cresyl violet). The sections were then digitized using a flat-bed scanner with resolution of 42 μm/pixel. As every 10th section was digitized, to meet both the current histological standards and the requirements for both multi-stain approaches and the possible merge with whole brain MR images, the digitized histological sections then were inflated to a virtual slice thickness of 200 μm, leaving the in-plane resolution untouched for maximum exploitation of the full histological information.

**Figure 2:**
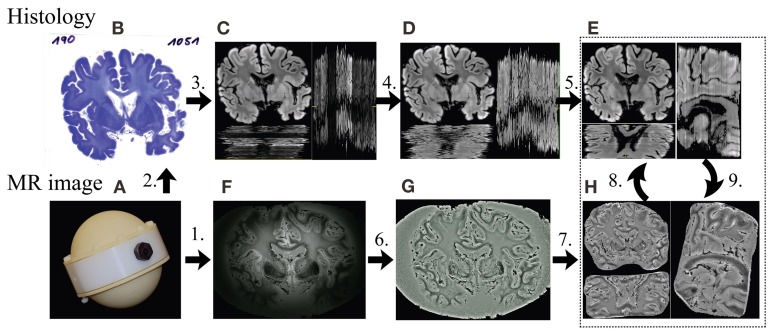
**Framework of the MRI-guided histology reconstruction.** 1. MRI measurement **(F)** with the container **(A)** at 7T; 2. Standard processing of histology and staining **(B)**; 3. Converting color to gray, removing the background **(C)**; 4. Inter-section intensity normalization using histogram matching **(D)**; 5. Inter-section rigid registration **(E)**; 6. Intensity correction using Gaussian filter **(G)**; 7. Removing of noisy background **(H)**; 8. Affine registration from MR image to histological 3D volume; 9. Non-linear registration from histology **(E)** to MR image **(H)** slice by slice.

The second post mortem human brain (73-year-old female), denoted as HB2, was fixed *in situ* by formalin injection (10% Histofix; Carl Roth GmbH + Co KG) into the left and right internal carotid arteries. After 24 h the brain was removed from the skull and immersed in Histofix 10% and stored in formalin. The brain was scanned under optimized conditions using a custom-built plastic sphere container (Figure 2A), which was built from two half hemispheres on both sides and a cylinder in the middle. The container was filled with formalin. To reduce the hotspots observed in the previous 7T images, formalin with higher concentration (7%) was used.

To increase field of view (FOV) and SNR, data were acquired with a 32 channel coil using a 3D gradient echo sequence with the following parameters: *TR* = 16 ms, *TE* = 5 ms, flip angle = 14°, bandwidth = 70 Hz/pixel, 150 μm isotropic resolution, matrix size = 1280 × 1280 × 1024, 1 average and total acquisition time = 5.7 h. The 230GB MRI raw data were reconstructed offline on a workstation (4 × Six-Core AMD Opteron™ processors, 2.8 GHz, 128 GB RAM) using a dedicated MATLAB^®^ program. This processing solution was utilized because the size of the dataset was beyond the computational capability of the built-in computer on the scanner. SNR is inversely proportional to resolution and— relative to conventional acquisitions—is much lower with the submillimeter resolution utilized in the present study. The image quality could be dramatically deteriorated by the noise if the multi-channel reconstruction algorithm based on sum-of-squares was used. Therefore, an adaptive image reconstruction optimized for low SNR (Walsh et al., 2000) was employed to combine the signals collected through the 32 channels.

HB2 was further scanned using a 3T whole body MRI scanner (Magnetom Trio, Siemens, Germany, Erlangen) and was acquired using Magnetization-Prepared Rapid Acquisition with Gradient Echo (MPRAGE) with the following parameters: *TR* = 2500 ms, *TE* = 3.47 ms, *TI* = 1100 ms, flip angle = 8° and resolution = 600 μm (isotropic) during the process of mask generation (see below). The resulting ultra-high resolution image was required to be down-sampled to 300 μm isotopic to pass the effective 32-bit SPM 8, which was fixed just very recently with SPM12. The intensity inhomogeneity of the brain image was corrected using an additional N4 bias field correction with four iterations (100 × 100 × 100 × 100), as provided by ANTs (Avants et al., 2011). The brain image was segmented using the VBM 8 toolbox implemented in SPM 8 with 100 mm FWHW for the bias field correction and without skull stripping. This was preferred toward using the DARTEL registration outside the VBM 8 toolbox.

### MRI-guided histology reconstruction

Using the techniques illustrated in Figure 1, the two human brain datasets were processed and the key steps are as follows (Figure 2):

1. MRI measurement in self-built container (Figure 2A) with ultra-high spatial resolution at 7T.
2. Standard processing of histology and staining (Figure 2B).
3. Color histological images (Figure 2B) were converted to 8 bits gray-scale images (Figure 2C) using equal weightings on each color channel for registration. The noisy background of each image was removed by applying a mask created using morphological operations, including eroding, dilating and connectivity analysis (Gonzalez et al., 2009). To reduce the computational time and memory consumption, the histological images used for MR coregistration were down-sampled by a factor of 2 using bilinear interpolation, resulting in an image matrix size of 2058 × 1536, with a pixel size of 85 × 85 μ m^2^ after prior estimations of computation time.
4. Inter-section intensity inhomogeneity of the histological images was corrected using histogram matching (Gonzalez et al., 2009) with one section, which was manually selected based on relative smooth intensity variation of staining and good contrast of structure as a reference to obtain a new set with homogeneous intensities (Figure 2D).
5. The rigid transformation between any two adjacent histological sections was obtained using the ANTs (Avants et al., 2011) with Normalized Mutual Information (NMI) as the cost function. A multi-pass registration strategy was taken to deal with the large difference in the initial positions of the image pairs in some cases. In each pass, different sets of parameters of ANTs were used. The transformations found in all passes were concatenated for calculating the registered image to avoid resolution loss caused by multiple interpolations. By composing the transformations between adjacent images, the series of 2D sections were stacked together to form an initial guess of the 3D histological volume (Figure 2E), to which the MRI volume was registered using affine transform.
6. As a pre-processing step on the MR image, a Gaussian filter based intensity inhomogeneity correction method for 7T MRI (Yang et al., 2009) was employed (Figure 2G). In this study, a Gaussian filter with Full Width at Half Maximum (FWHM) of 5 mm was used.
7. The noisy background of the MR image HB1 was removed (Figure 2H) manually slice by slice using an open source software MIPAV (MIPAV, 2012). Given the improvements offered by the introduction of a 3T mask, this was not necessary for HB2.
8. The MRI volume was registered using affine transform to the histological volume. The registered MRI volume was re-sliced in the histological space using MIPAV and the corresponding MRI slice for each histological section was found (Figure 2H).
9. The histological sections were then non-linearly registered to their corresponding MRI slices using ANTs and stacked to obtain an updated histological 3D volume, to which the MR volume was aligned again.

Steps 8 and 9 were repeated until there was no significant improvement in the similarity, measured by NMI, between the MRI volume and the histological volume. The transformation matrix was then applied to the histological sections with the original spatial resolution.

## Results

### Results on HB1

Sample histological sections before (Figure 2B) and after (Figure 2C) noisy background removal are shown in Figure 2. A comparison of the histological reconstruction both with (Figure 2C) and without (Figure 2D) intensity correction is illustrated. It can be observed that the cross section intensity correction of histological sections led to highly improved homogeneity for image intensities (Figure 2D).

**Figure 3:**
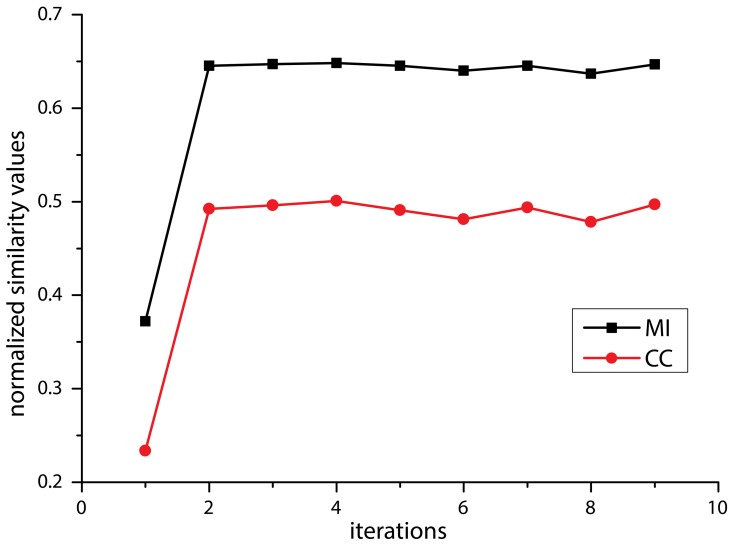
**Diagram of mean value of similarity value change using Normalized Cross Correlation (NCC, red) and Normalized Mutual Information (NMI, black) against the number of affine registration**.

To determine the number of passes needed to achieve stabilized inter-section rigid registration, the mean similarity value changes quantified by NMI and Normalized Cross Correlation (NCC)—measured across 15 random adjacent histological section pairs—was plotted against the number of passes, as shown in Figure 3. Insignificant improvement or smaller similarity values were observed after 4 passes. Consequently, a maximum of 4 iterations were performed in the multi-pass inter-section rigid registrations.

**Figure 4:**
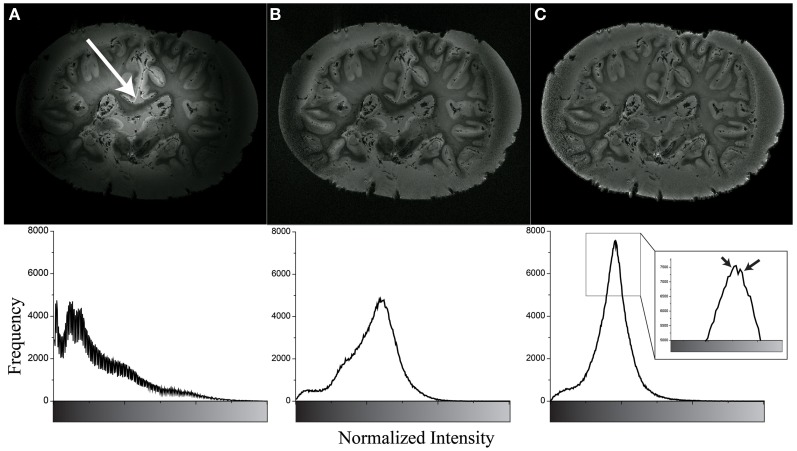
**Example of inhomogeneity correction of original image (A) using N4 bias field correction (B) and Gaussian filter based method (C).** The bottom row shows their intensity histogram. The white arrow shows the center of hotspot. The black arrows show WM signal (left) and GM and formalin signal (right). Note that the high border signals in panels (**B** and **C**) are know effects of the N4 algorithm, however, these borders were removed before further image processing.

As shown in Figure 4, the Gaussian-filter-based intensity correction yielded sufficient correction for the strong intensity inhomogeneity of the MR image by visual inspection. Quantitatively, the Gray-Level Co-occurrence Matrices (GLCM) (Haralick et al., 1973) indicated an improvement from 0.8382 to 0.9951, implying that better homogeneity was achieved. In comparison, N4 bias field correction (Tustison et al., 2010) was slightly less efficient in improving homogeneity (GLCM = 0.9810). In both methods, the hotspots, i.e., foci of localized high, but radially decreasing intensities in the image could be effectively removed and the outer brain regions sufficiently recovered. The zoom-in window in Figure 4C shows the different intensity distributions of WM signal and Gray Matter (GM) with formalin background signal.

**Figure 5:**
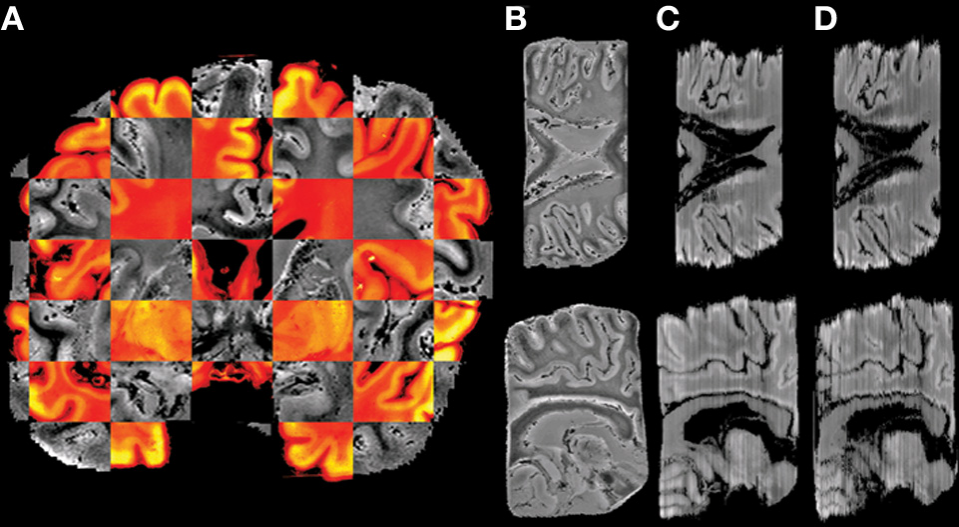
**Example of histology reconstruction of HB1. (A)** Checker-board view of affine registered MR slice (gray) and histological section (color) showing good cross-modality alignment in the fine structures. The axial and sagittal views of the images are shown in the top and bottom row, respectively. **(B)** MR volume in histological space after 3D affine registration, **(C)** Histological volume reconstructed by section-to-section 2D affine registration, and **(D)** Histological volume created by stacking the non-linearly registered histological sections to the corresponding MR slices.

A co-registered histological section and MR slice pair is shown as a checker-board view in Figure 5A. The continuity in the fine structures in the pictures indicates that suitable alignment was achieved across the two imaging modalities (Figures 5B,C).

**Figure 6:**
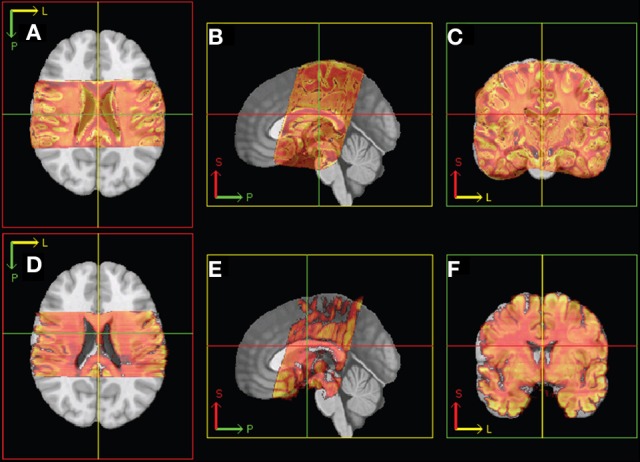
**Illustration of linear registration from MR (top: A–C) and histology (bottom: D–F) 3D volume to T1 template.** Axial (left), sagittal (middle), and coronal (right) views. P, posterior; S, superior; L, left.

The subsequent non-linear (2D) registration of individual histological sections to their corresponding coronal MR slices did not improve the similarity achieved in step 6. In direct comparison, NMI of neighboring histological sections dropped from 0.65 to 0.55 on average (Figure 5D). Therefore, transformation of histological sections into MR space was performed using the inverse affine transformation matrix from step 6 only. Then spatial normalization of histological sections into a standard anatomical space was successfully performed by applying the affine/non-linear transformation generated by normalizing the MR source data into T1 template MNI152 Space (BIC, 2009) (Figure 6).

### Results on HB2

**Figure 7:**
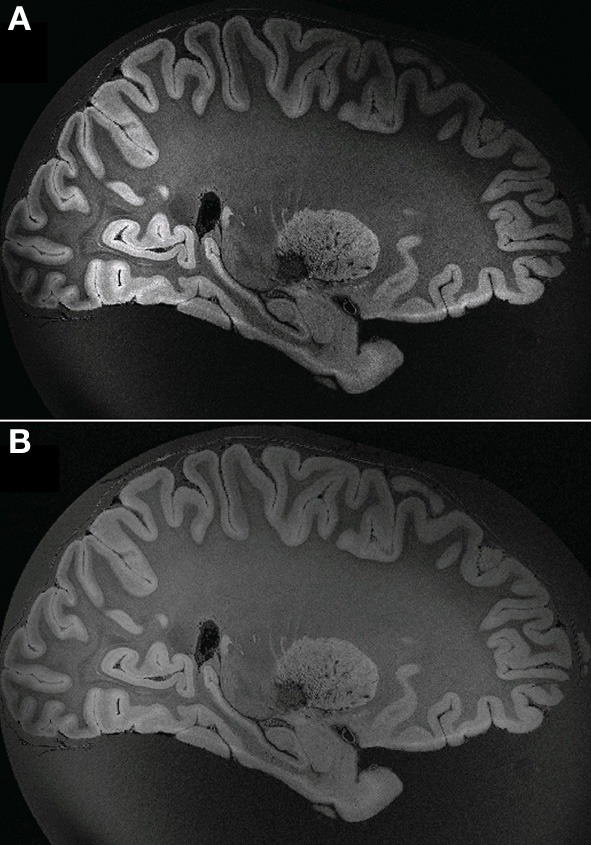
**Reconstruction comparison of sum-of-squares (A) and adaptive reconstruction (B)**.

**Figure 8:**
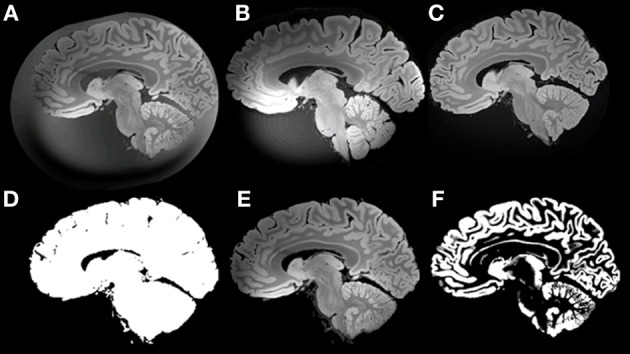
**Example of masking of the 7T ultra-high resolution data set. (A)** ultra-high resolution data set; **(B)** MPRAGE at 7T; **(C)** MPRAGE at 3T; **(D)** Mask; **(E)** masked ultra-high resolution data; **(F)** GM after Segmentation using VBM8.

**Figure 9:**
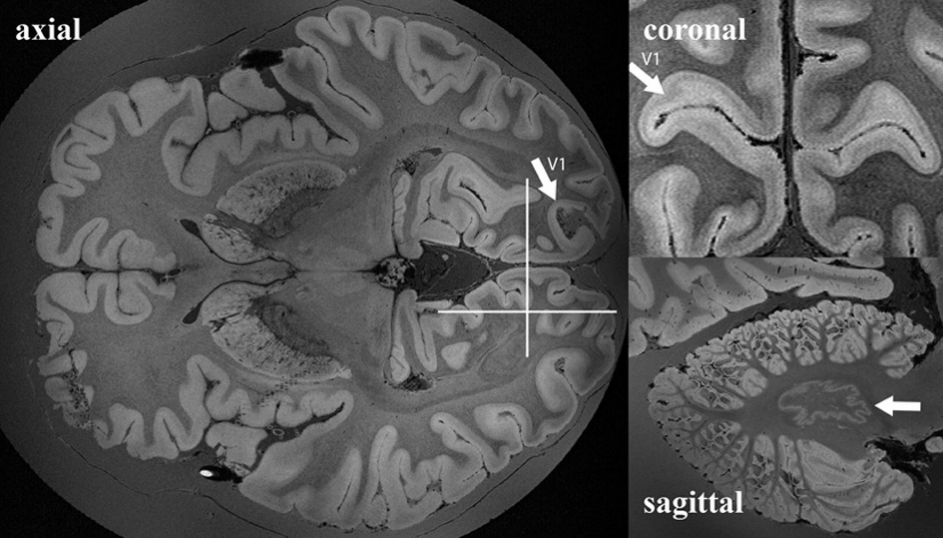
Example of whole *ex vivo* brain (HB2) 3D gradient echo T2^*^-weighted imaging with 150 μm isotropic resolution. White arrows in the axial and coronal views point to the primary visual cortex. White arrows in the sagittal view point to nucleus dentatus of the cerebellum. White lines in the axial view on the right show the anatomical locations of the coronal and the sagittal views on the left.

The sub-optimal non-linear registration and difficulties in automatic background removal for the MR image of HB1, which in principle was shown to be feasible in the mouse brain, was most likely due to the insufficient background contrast on the post mortem human brain. We thus applied a modified MR acquisition and reconstruction scheme on the second brain scanned (HB2). As shown in Figure 7, the adaptive reconstruction algorithm led to increased SNR for WM (*ca*. 1.3) and GM (*ca*. 1.7) compared to the sum-of-squares algorithm. To further facilitate segmentation, brain masks were compared for their feasibility. Both MPRAGE and gradient echo at 7T suffered from severe intensity inhomogeneities and low brain-formalin contrast (Figures 8A, B). In contrast, the 3T MPRAGE image (Figure 8C) provided a sufficiently homogeneous formalin background with relatively low signal intensity, which could be easily removed by thresholding to create a brain mask (Figure 8D). After registering the 3T and 7T images of the same brain, this mask could thus be used to remove the formalin background for the high-resolution 7T image (Figure 8E). These steps then made a subsequent automated segmentation of the ultra-high resolution anatomical MR image possible (Figure 8F). Furthermore, HB2 with higher SNR and spatial resolution provided a better opportunity to discern anatomical substructures such as the stria of Gennari (V1) or cerebellar nuclei (Figure 9).

## Discussion

### Histology reconstruction

Previous work on mice demonstrated the feasibility and strength of histological volume reconstructions relying on guidance by high-resolution structural MRI of the same brain (Yang et al., 2012b). The lost information about the spatial relationships between adjacent histological sections could be restored using 3D MR volumes and the non-linear tissue distortions could be thereby corrected. The result is a pair of histological and MR volumes of the same brain in the same spatial reference space, providing integrated information for morphometrical analysis in 3D. For the first time, we were able to adapt the same technique to fixated human brains, guided by 7T high-resolution MRI volumes. Although this has not been empirically demonstrated, a recent study suggests that blockface imaging (Amunts et al., 2013) may also be able to achieve the resolution of histological volumes (200 μm in the z direction), as we have shown in this investigation. However, high-resolution (150 μm) 7T MRI has the additional advantages that it is able to measure different MRI contrasts using different acquisition parameters, and does not require extra equipment or handling for taking blockface images. Adaptation of the method we applied to the mouse brain to human specimens worked without major difficulties. However, we encountered novel technical problems for human brain imaging at 7T, particularly with respect to intensity inhomogeneity and formalin background issues. By solving these problems we were able to assure that MR image quality is sufficient for the proposed histology reconstruction algorithms. Not only could we generate a 3D volume out of digitized histologically stained brain sections (a video demonstration is available in Supplementary material as well as at the following URL: http://www.canlab.de/video/3DHistology.html), but this method was also the first step for MR-guided normalization of histological information into a 3D standard space, ultimately allowing for co-localization areas from multiple post mortem brains or different stains for the same brain (Figure 9).

Besides the preparatory issues such as inhomogeneous stains, folding and split parts (Ju et al., 2006) that we encountered in reconstructing mouse brain histological data, we faced particular challenges at high field imaging data of human brains: not only is the B_1_-field at 7T is more than half of the length at 3T, and more than ¼ of the length at 1.5 T, but the MRI intensity inhomogeneity is also double and 4 times stronger than at 3T and 1.5T (Ibrahim et al., 2001). This necessitated us to develop an adapted image reconstruction and to apply a Gaussian filter in order to obtain sufficient recovery of brain information.

The successful reconstruction of the histological sections was possible after using corrections based on histogram matching of intensities from imperfect staining or suboptimal digitization by the flat-bed scanner. The intention to develop an automated pipeline was successfully realized except for step 5, due to the pre-processing problem in automatically removing the formalin background, which has similar intensities at 7T. While this step initially required manual segmentation, a solution was therefore developed using 3T MPRAGE images of the same brain, which provided an effective and feasibly applied brain mask for removing the formalin background in a 7T image. Further benefits of the additional modification of the acquisition scheme could be reached by adapting the image acquisition parameters, resulting in superb anatomical resolution while reducing the acquisition duration from 40 to 6 h.

Such improvements of MR data quality are crucial, especially for the success of deformable registrations, which would allow for correction of tissue-specific shrinkage during the subsequent histological preparations. Indeed, without successful segmentation, non-linear registration performed slightly worse than rigid body registration of histological sections, while the latter was at least feasible for affine normalization of the histological volume into standard MR space. Furthermore, we showed that 3D reconstruction was feasible for partial brain blocks, adding practical relevance to the work of histological laboratories incapable of preserving whole brains, especially as is usually the case in patients, or if only blocks of main interest were to be sectioned for a given observation (Figure 6).

In addition to modifications of the image acquisition and reconstruction, we also observed a potential influence of the fixation method. For the second brain (HB2) we chose a formalin concentration of 7%, given that it reduced the occurrence of signal hotspots as compared to 3.5%. Compared to Figure 8B, the hotspot in Figure 4A was much stronger such that the signal at the boundary was nearly zero. Automatic removal of the formalin background further relied on optimization of the inversion recovery sequence for a specific inversion time, with calculations based on formalin T1 values to suppress this signal selectively for mask generation at 3T.

### Ultra-high resolution imaging

This study also demonstrated the feasibility of MR microscopic imaging of fixated entire brains. *Ex vivo* imaging of one hemisphere has already been shown with resolutions up to 100 μm isotropic (Iacono et al., 2011). However, microscopic MR imaging of large samples is limited by achievable SNR, as well as practical factors such as immense raw data sizes, causing problems in image reconstruction and post-processing. To the best knowledge of the authors, this is the first demonstration of achieving an isotropic resolution as detailed as 150 μm for the entire human brain. This technique offers structural investigation on a microscopic scale of fixated brains, even if they must be preserved whole. However, significant decreases occur in SNR when increasing spatial resolution of MR images, limiting the potential exploitation of the improved resolution by a power of 3. Nonetheless, the image quality achieved in this study successfully addresses this problem with an adaptive multi-channel image reconstruction algorithm that utilized principle component analysis. Compared to the conventional reconstruction algorithm based on sum-of-squares, our method not only improved SNR by a factor of as much as 1.7, but we were also able to achieve improved homogeneity. Although the image of HB2 was acquired with a single average, it shows acceptable SNR (*ca*. 50) in comparison to HB1 scanned with 10 averages with SNR ranging from 10 to 80.

It should be noted that, in comparison to *in vivo* conditions, the formalin-fixated brain has lower T1 values. Thus, the gradient echo signal is higher than that found *in vivo*. This is an additional factor contributing to the feasibility to measure with such high resolutions. In the future, with more averages, the resolution could thus be expected to reach or even pass 100 μm (isotopic) by keeping comparable SNR.

Along with SNR and the prolonged acquisition duration, large data sizes and heavy computational burdens represent additional challenges to be tackled. In this study, it took 5.7 h to acquire MR images with 150 μm isotopic resolution, requiring a 7T scanner and generating 230 GB raw data. It took 25 days to process the raw data offline using adaptive reconstruction in MATLAB^®^ on an advanced server with 24 CPUs and 128 GB physical memory. By increasing the resolution, acquisition time is extended, and data size increases cubically. Therefore, the adaptive reconstruction algorithm proposed here might need to be implemented with extensive physical memory and parallel computing approaches, or using general-purpose GPUs (Amunts et al., 2013). These limitations make the necessity of multi-disciplinary collaborations obvious. Ultra-high-field scanners are still a rarity; however, the fixed specimen can be transported to suited centers for MR scanning, while computational demands can be tackled by using larger accessible clusters. Likewise, the use of whole brain microtomes is another methodological hurdle to be tackled, which may require additional collaborations with centers providing experienced staff.

One may hope that the method described here might offer benefits that may reduce the necessities of *in situ* fixation. Indeed, this would strongly depend on the amount of additional deformation after *ex situ* preparation. Since we did not directly test this matter, the extent of these benefits remain an open question for dedicated follow-up research, challenging the specific gains of MR-based 3D reconstruction and normalization.

### Histology vs. ultra-high resolution MRI

Accurate registration of histology and MR images facilitates the analysis of complementary information from the two modalities. For example, histological volumes reconstructed utilizing the method described herein were used to evaluate track-density imaging (TDI), a novel high-resolution technique for diffusion MRI (Calamante et al., 2012). For example, MR-based fiber tractography might be addressed by polarized light imaging of histology (Axer et al., 2011). At 360 μm isotropic resolution, identification of thalamic nuclei becomes evident given the high SNR at sub-millimetre resolutions. Similarly, the delineation of individual neocortical layers of the hippocampus appeared, while resolutions still limited the estimation of folding curvatures such as those observed in the hippocampus (Ekstrom et al., 2009).

**Figure 10:**
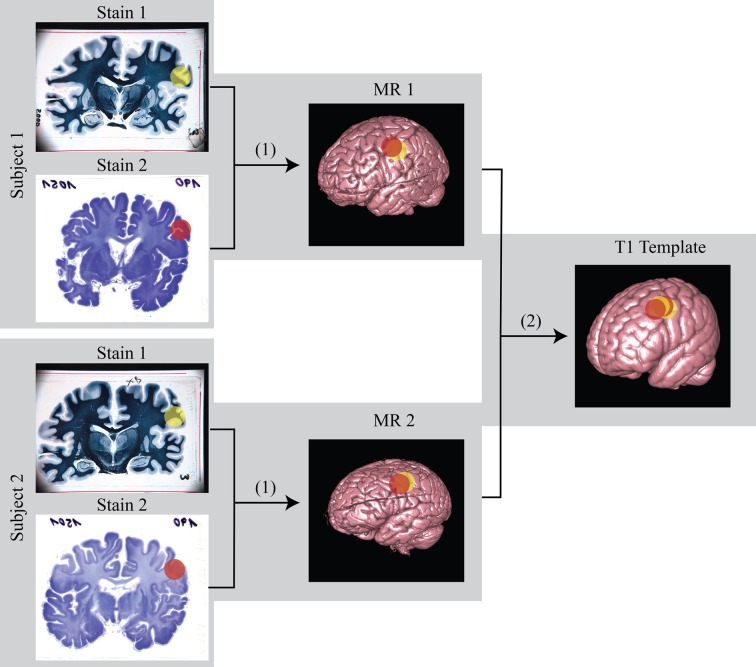
**Schematic diagram of the registration from histology to MR and then to standard template.** (1) linear/non-linear registration from histology space to MR space; (2) non-linear registration from MR space to standard template space. Using this transformation matrix, histology can be transformed to template space as well. The color sphere labels mark the region of interest (ROI).

With a thickness of 150 μm in MRI, we reached the effective through-plane resolution usually only achievable by histology sections, given that many sites either keep only one out of ten adjacent sections of normally 20 μm. Even if every consecutive section were preserved, this would be insufficient for achieving the maximum of 20 μm as long as multiple stains were intended. The in-plane resolution of MRI, 150 μm, also approached the level of digitized histology sections using a flat-bed scanner (42 μm). In comparison to Figures 4, 9 shows that ultra-high resolution MR images have the potential to explore more fine structure details similar to those identified in the histological sections. Using these details in conjunction with MR images could improve registration results and correct non-linear tissue distortions in histology sections better than before. Most importantly, histology sections of multiple stains can now be registered to a common space (Figure 10), allowing combined overlays of several immune histochemical observations, even across subjects. The successful 3D reconstruction of such histological information of multiple individuals provides grounding for whole brain analysis strategies similar to methods applied for structural and functional MRI. Notably, Lüsebrink et al. (2013) have reported a decrease of estimated cortical thickness acquired by MRI along with increasing of spatial resolution at 1.0 and 0.5 mm due to partial volume effects (Lüsebrink et al., 2013). Our new ultra-high resolution MRI of the entire human brain provides further opportunities for investigating these sort of changes and identifying the most optimal spatial resolution of the 3D-MR morphometry for future clinical studies. Excitingly, 3D based analysis of cortical thickness derived from histological contrasts may represent a crucial covariate to interpret cellular microscopic differences in the context of local thicknesses (Wähnert et al., 2010), thereby providing a potential “gold standard” for measurement.

## Conflict of interest statement

The authors declare that the research was conducted in the absence of any commercial or financial relationships that could be construed as a potential conflict of interest.

## Supplementary material

A 3D-rendered video is available: http://www.frontiersin.org/Neuroanatomy/10.3389/fnana.2013.00031/abstract
